# Optimizing Crop Yield Prediction: An In-Depth Analysis of Outlier Detection Algorithms on Davangere Region

**DOI:** 10.1155/tswj/9312639

**Published:** 2025-06-29

**Authors:** C. S. Anu, C. R. Nirmala, A. Bhowmik, A. Johnson Santhosh

**Affiliations:** ^1^Department of Computer Science and Engineering, Bapuji Institute of Engineering and Technology, Davangere, Karnataka, India; ^2^Visvesvaraya Technological University, Belagavi, Karnataka, India; ^3^Department of Additive Manufacturing, Mechanical Engineering, SIMATS, Saveetha Institute of Medical and Technical Sciences, Chennai, India; ^4^Division of Research and Development, Lovely Professional University, Phagwara, Punjab, India; ^5^Faculty of Mechanical Engineering, Jimma Institute of Technology, Jimma, Ethiopia

**Keywords:** elliptic envelope, iterative R, one-class SVM, outlier, random forest, SMVOD, SSVD

## Abstract

Crop yield prediction is a critical aspect of agricultural planning and resource allocation, with outlier detection algorithms playing a vital role in refining the accuracy of predictive models. This research focuses on optimizing crop yield prediction in the Davangere region through a thorough analysis of outlier detection algorithms applied to the local agricultural dataset. Six prominent algorithms, including isolation forest, elliptic envelope, one-class SVM, iterative R, spatial singular value decomposition (SSVD), and spatial multiview outlier detection (SMVOD), are systematically evaluated. The study emphasizes the significance of accurate crop yield predictions in local agriculture and assesses each algorithm's performance using precision, recall, accuracy, and *F*1 score metrics. Elliptic envelope demonstrates its efficacy in handling the unique characteristics of the Davangere dataset. This method demonstrated improved performance in refining the crop yield prediction model by identifying and removing outliers, thereby contributing to more accurate predictions and optimized planning in the dynamic landscape of the Davangere region.

## 1. Introduction

Modern agriculture is undergoing a transformative phase with the integration of cutting-edge technologies, data analytics, and machine learning. The ability to accurately predict crop yields is crucial for sustainable farming practices, resource optimization, and ensuring food security [1–4]. In this context, the application of outlier detection algorithms has emerged as a promising avenue for refining crop yield prediction models. This paper delves into the intricacies of outlier detection within the realm of precision agriculture, focusing on a case study conducted in the Davangere region—an agriculturally significant area in Karnataka, India [5–8].

The Davangere region, known for its diverse agricultural landscape, presents unique challenges that demand tailored outlier detection techniques. Traditional methods often struggle to address the complexities inherent in agricultural datasets, characterized by varying soil conditions, climate dynamics, and crop types [9–12]. Hence, this study explores the efficacy of six advanced outlier detection algorithms: isolation forest, elliptic envelope, one-class support vector machine (one-class SVM), iterative R, spatial singular value decomposition (SSVD), and spatial multiview outlier detection (SMVOD) [5]. By applying these algorithms to the locally curated dataset, we aim to scrutinize their performance in identifying anomalies that could significantly impact crop yield predictions.

In addition to evaluating the algorithms individually, we applied a variant of the elliptic envelope approach, which combines principal component analysis (PCA) with density-based spatial clustering of applications with noise [3, 13–16]. This method is specifically designed to address the intricacies of the Davangere dataset, aiming to enhance outlier detection and improve the overall accuracy of crop yield predictions. The outcomes of this study not only contribute to the growing body of knowledge in precision agriculture but also hold practical implications for farmers, policymakers, and stakeholders involved in agricultural decision-making in the Davangere region and similar agroecological contexts [17–21]. This research strives to provide valuable insights for sustainable farming practices in the face of evolving environmental and climatic conditions.

## 2. Literature Review

Arun Kumar et al. proposed the spatial iterative R algorithm, emphasizing its efficiency in identifying outliers and mitigating the impact of masking and swamping effects on the dataset [1]. This iterative approach holds promise in addressing the challenges associated with outlier detection, especially in scenarios where anomalies might be overshadowed by prevalent data patterns [1].

Zhao et al. introduced SMVOD, a method that leverages spatial information from multiple views to comprehensively capture outliers. This approach acknowledges the multidimensional nature of data and strives to enhance outlier detection by considering diverse aspects simultaneously [2].

Ma and Yin focused on PCA as a novel method for large-scale and time-dependent data analysis. The use of robust techniques suggests a dedicated effort toward addressing challenges posed by outliers and anomalies within the data, particularly in the context of dynamic and voluminous datasets [3].

Xu et al. explored the isolation forest algorithm, introducing a new representation scheme involving randomly initialized neural networks. This innovative approach is aimed at enhancing data partitioning through random axis-parallel cuts, showcasing a creative adaptation of neural network–based techniques for outlier detection [4].

Arun Kumar et al. provided a comprehensive analysis of spatial multivariate outlier detection methods. Their study not only explores existing methods but also sheds light on their strengths and limitations, contributing to a nuanced understanding of spatial multivariate outlier detection techniques [5].

Nasir Usman et al. conducted an analysis using elliptic envelope, isolation forest, and one-class SVM. Notably, they underscored the impact of hyperparameter tuning on the models' performance, showcasing improvements in metrics such as precision, recall, specificity, accuracy, AUC, and *F*1 score, particularly for isolation forest [6].

Liu et al. proposed PCA-DBSCAN, addressing practical challenges such as deviations from spectrometers and anomalies in spectrum characteristic peak intensities. This approach highlights the often-overlooked presence of outliers in categorical data, offering a novel perspective on outlier detection in specific domains [7].

Collectively, these studies contribute to the evolving landscape of outlier detection methods, offering valuable insights and innovative approaches tailored to diverse datasets and application domains. The diverse methodologies and considerations presented in these works provide a rich foundation for further advancements in the field of outlier detection.

## 3. Dataset Collection and Description

For this research, we collected an extensive agricultural dataset from Taralabalu Krushi Vignyana Kendra, Davangere, capturing crucial parameters that significantly influence crop yield prediction. The dataset encompasses diverse features, providing a holistic view of the agricultural landscape in the Davangere region [8, 22–25]. The dataset was locally sourced, ensuring its relevance to the specific agroecological conditions prevalent in the area.

### 3.1. Features

1. N (nitrogen) represents the soil N content, a vital nutrient for plant growth.
2. P (phosphorus) indicates the soil P levels, essential for root development and energy transfer.
3. K (potassium) reflects the soil K content, crucial for overall plant health.
4. pH measures the acidity or alkalinity of the soil, influencing nutrient availability.
5. Temperature represents the ambient temperature, impacting crop growth and development.
6. Rainfall indicates the amount of precipitation, a key factor in water availability for crops.
7. Area denotes the cultivated area for a specific crop, influencing yield calculations.
8. Production represents the total agricultural production for the specified crop.
9. Yield signifies the crop yield, the primary target for prediction in this study.
10. Season categorizes the time of cultivation, considering factors like monsoon and winter.
11. Lat (latitude) provides the geographical Lat of the cultivation area.
12. Long (longitude) specifies the geographical Long of the cultivation area.
13. Soil describes the soil type, a critical factor in crop suitability and nutrient availability.

### 3.2. Dataset Collection Procedures

The data collection process involved collaboration with Taralabalu Krushi Vignyana Kendra, Davangere, a local agricultural research center. Soil samples were obtained from various farms in the Davangere region, covering multiple crops and cultivation practices. Additionally, weather data, including temperature and rainfall, was sourced from local meteorological stations. The dataset was meticulously curated to ensure representativeness across different seasons and crops, providing a comprehensive snapshot of agricultural conditions in the region [26–31].

### 3.3. Dataset Description

The dataset comprises a structured collection of records, each representing a specific instance of agricultural cultivation in the Davangere region. It includes numerical values for soil nutrients (N, P, and K), pH levels, temperature, rainfall, cultivated area, production, and yield. Categorical features such as the season of cultivation, soil type, and geographical coordinates (Lat and Long) are also incorporated [32–35]. This rich and diverse dataset serves as the foundation for evaluating the efficacy of outlier detection algorithms in enhancing crop yield prediction accuracy, contributing valuable insights to precision agriculture practices in the Davangere region.  Table 1 shows the sample records collected.

## 4. Outlier Detection Algorithm

This part delves into the pivotal role of outliers in influencing the precision and dependability of agricultural predictions. We embark on an exploration of diverse outlier detection and handling techniques aimed at fortifying the resilience of our crop yield prediction models. The arsenal of methods enlisted comprises isolation forest, DBSCAN, one-class SVM, elliptic envelope, iterative R, SSVD, and SMVOD. Each of these methods offers a distinctive approach to discerning and mitigating outliers, thereby enriching our comprehension of the dataset and elevating the efficacy of predictive models [36–39].

### 4.1. Isolation Forest

Isolation forest is an algorithm used for outlier detection, particularly in high-dimensional datasets [4–6]. The basic idea behind the isolation forest is to isolate anomalies by recursively partitioning the data until the anomalies are isolated in small partitions. The algorithm is based on the observation that anomalies are typically few and far from the normal instances. Here is a simplified explanation of the isolation forest process and the associated equation.

#### 4.1.1. Equations and Methodology

i. Random selection of features and splitting

At each iteration, a random feature is selected.

A random split point along that feature is chosen.

ii. Recursive partitioning

The data is split into two subsets based on the selected feature and split point.

This process is repeated recursively until anomalies are isolated into small partitions.

iii. Scoring

Anomalies (outliers) require fewer splits to be isolated, and their average path length to reach isolation is shorter.

iv. Normalization

The path lengths are normalized to obtain an anomaly score.

The equation for isolation forest is as follows.

The average path length for a data point *X* in the isolation forest is given by the formula

(1) $$\begin{array}{c} h\left(X\right)=E\left(h\left(X\right)\right) \end{array}$$

where *h*(*X*) is the path length for data point *X* and *E*(*h*(*X*)) is the expected path length.

### 4.2. Elliptic Envelope

**Figure 1:**
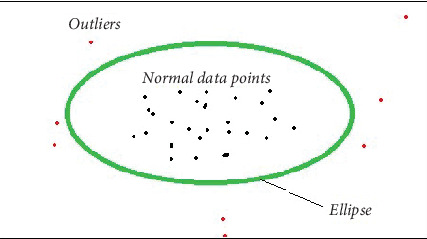
Conceptual diagram of the elliptical envelope.

The standard elliptic envelope algorithm is used for robustly estimating the covariance of a dataset and identifying outliers. It assumes that the majority of the data follows a Gaussian distribution, and it models the inlying data points as an elliptical-shaped envelope which is shown in Figure 1. Outliers, which deviate significantly from the assumed distribution, are identified based on their Mahalanobis distances [6].

In the diagram, the green area is an ellipse. Therefore, an imaginary elliptical area is created around a given dataset by the elliptical envelope algorithm. Any values outside of the envelope are returned as outliers, while values inside the envelope are regarded as typical data. Naturally, this algorithm should recognize the red data points in the above diagram as outliers. Figure 1 makes it clear that a Gaussian distribution of the data is ideal for the algorithm to function.

Mahalanobis distance is a measure of the distance between a point and a distribution, taking into account the correlation between variables. It is defined as

(2) $$\begin{array}{c} D_{M}\left(x\right)=\sqrt{\left\{{\left(x-\mu \right)}^{T}\Sigma^{\left\{-1\right\}\left(x-\mu \right)}\right\}} \end{array}$$

where *x* is the data point, *μ* is the mean of the distribution, and Σ is the covariance matrix of the distribution.

The elliptic envelope fits around the inlying data points, assuming a Gaussian distribution. The envelope is defined as

(3) $$\begin{array}{c} {\left(x-\mu \right)}^{T}\Sigma^{\left\{-1\right\}}\left(x-\mu \right)\leq \chi^{2}\alpha \end{array}$$

where *X*^2^_*α*_ is the chi-square threshold for a given confidence level *α*.

The proposed elliptic envelope uses PCA and Euclidean distance instead of Mahalanobis distance. PCA transforms the data from a high-dimensional space into a lower-dimensional principal component (PC) space:

(4) $$\begin{array}{c} X^{\prime }=XW \end{array}$$

where *X* is the original data matrix (*n* × *d*) and *W* is the matrix of PCs (eigenvectors of the covariance matrix). *X*′ is the transformed data in PC space (reduced dimensions), Euclidean distance for outlier detection. Instead of using Mahalanobis distance, the proposed method computes the Euclidean distance from the center:

(5) $$\begin{array}{c} D_{E}\left(x\right)-\sqrt{\left\{{\left(x^{\prime }-\mu^{\prime }\right)}^{T}\left(x^{\prime }-\mu^{\prime }\right)\right\}}. \end{array}$$

Instead of assuming an elliptical distribution, the proposed method determines a threshold dynamically:

(6) $$\begin{array}{c} D\_E\left(x\right)>P\_\left\{95\right\} \end{array}$$

where *P*95 is the 95th percentile of all distances. If a point is farther than this threshold, it is classified as an outlier.

The key difference between the proposed elliptic envelope and standard elliptic envelope is that the standard elliptic envelope fails on skewed or non-Gaussian data, which real-world agricultural datasets often have, and it also assumes an elliptical boundary, which is not flexible for complex data distributions, whereas the proposed elliptic envelope used PCA and Euclidean distance, which are more flexible because PCA captures the major variance, reducing noise from irrelevant features. Euclidean distance is simpler and works better for arbitrary-shaped distributions: more robust for high-dimensional data, where Mahalanobis distance struggles.

### 4.3. One-Class SVM

One-class SVM is a machine learning algorithm used for anomaly detection, particularly in situations where the majority of the data belongs to one class (normal) and anomalies (outliers) are rare. One-class SVM is aimed at find a hyperplane that separates the normal instances from the origin in the feature space [6, 40–42].

#### 4.3.1. Equations and Methodology

##### 4.3.1.1. Linear One-Class SVM

In the linear case, the objective function for one-class SVM can be written as

(7) $$\begin{array}{c} \min_{w,p}½.{\left\|w\right\|}^{2}–\rho \end{array}$$

where *w* is the weight vector, *ρ* is the offset from the origin, and *ϕ*(·) is the mapping of the input data to a higher-dimensional space.

##### 4.3.1.2. Nonlinear One-Class SVM

In the nonlinear case, the linear decision function is transformed into a nonlinear one using the kernel trick. The objective function becomes

(8) $$\begin{array}{c} \text{mi}{\text{n}}_{\begin{array}{c} \left\{w,\rho ,\xi \right\}1/2\\ . \end{array}}{\left\|w\right\|}^{2}-\frac{1}{vn}\overset{n}{\underset{\left\{i=1\right\}}{\sum }}\xi_{i} \end{array}$$

where *Ξi* are slack variables representing the classification error and *ν* is a parameter controlling the trade-off between maximizing the margin and minimizing the classification error.

One-class SVM is aimed at finding a hyperplane that encapsulates the normal instances in the feature space. The linear and nonlinear formulations depend on the problem's complexity, and the decision function is used to classify instances as normal or outliers based on their position relative to the hyperplane. The algorithm is parameterized by *ν*, controlling the trade-off between maximizing the margin and minimizing the classification error [34, 43–45].

### 4.4. Iterative R

Iterative outlier detection using the interquartile range (IQR) is a method for identifying outliers in a dataset by iteratively applying the IQR criterion. The IQR is a measure of statistical dispersion that is based on the range between the first quartile (Q1) and the third quartile (Q3) of the data. Outliers are typically defined as data points that fall below Q1 − *k*∗IQR or above Q3 + *k*∗IQR, where *k* is a user-defined threshold [1, 2, 46–50].

#### 4.4.1. Equations and Methodology

Here is how the iterative outlier detection using IQR works:

1. Compute initial IQR.
2. Calculate the initial IQR for the dataset. The IQR is given by: IQR = Q3 − Q1 where Q1 is the first quartile and Q3 is the third quartile.
3. Set threshold: Choose a threshold *k* to determine the range for identifying outliers. Common choices are 1.5 or 3.0, but the value depends on the desired sensitivity to outliers.
4. Identify outliers: Identify outliers by considering data points below Q1 − *k* × IQR or above Q3 + *k* × IQR.
5. Remove outliers: Remove identified outliers from the dataset.
6. Recompute IQR: Recalculate the IQR for the updated dataset.
7. Repeat: Repeat Steps 3–5 until no more outliers are found or until a predefined number of iterations is reached.

The iterative process involves updating the IQR in each iteration and re-evaluating the range for identifying outliers.

(9) $$\begin{array}{c} {\text{IQR}}_{\text{new}}=\text{Q}3_{\text{new}}-\text{Q}1_{\text{new}} \end{array}$$

where Q1_new_ is the new first quartile after removing outliers and Q3_new_ is the new third quartile after removing outliers. This iterative approach allows for the adaptability of the outlier detection process to the characteristics of the dataset, as removing outliers can affect the quartile values and, consequently, the IQR. The method is useful when the data distribution is not known in advance, and the iterative nature helps refine the outlier detection process over successive iterations [51–54].

### 4.5. SSVD

SSVD is a technique used for decomposition and dimensionality reduction of high-dimensional datasets, particularly in the context of spatial or spatiotemporal data. While SSVD itself is not designed specifically for outlier detection, it can be applied in combination with other techniques to identify outliers in certain scenarios. SSVD is often used for tasks such as feature extraction and noise reduction [5, 55–57].

SSVD is applied to decompose a data matrix *X* into three matrices *U*, *D*, and *V*, where

(10) $$\begin{array}{c} X\approx UDVT. \end{array}$$

Each of these matrices captures different aspects of the data, and the singular values in the diagonal matrix *D* represent the importance of the corresponding components.

While SSVD itself is not inherently designed for outlier detection, it can be used as a preprocessing step for outlier detection in spatial data. The idea is to identify outliers by examining the residuals or differences between the original data matrix *X* and its reconstructed approximation using the SSVD components.

### 4.6. SMVOD

SMVOD is a method designed to detect outliers in datasets that exhibit spatial structures, particularly when multiple views or aspects of the data are considered simultaneously. In the context of a crop yield prediction dataset with features such as N, P, K, pH, temperature, rainfall, cultivation area (AREA), production quantity (PRODUCTION), yield (YIELD), geographic coordinates (Lat, Long), soil type (SOIL), and season (SEASON), SMVOD can be employed to identify spatial outliers [8–14].

#### 4.6.1. Equations and Methodology

1. Let *X* represent the dataset with *n* samples and *m* features. Each sample is represented by *X*_*i*_ where *i* = 1, 2, 3, ⋯*n*. The features include both spatial (geographic coordinates) and nonspatial attributes.
2. Spatial structure integration: SMVOD integrates the spatial structure of the data by considering the spatial coordinates (Lat, Long). The spatial structure is incorporated through a spatial weight matrix *W*_*s*_ capturing the relationships between samples based on their geographical proximity.

(11) $$\begin{array}{c} W_{s}=\exp\left(-\left\{{\left\|D\right\|}^{2}\right\}/\left\{\sigma^{2}\right\}\right) \end{array}$$

where *D* is the spatial distance matrix between samples and *σ* is a bandwidth parameter controlling the influence of spatial distances.

3. SMVOD: SMVOD leverages the spatial weight matrix *W*_*s*_ along with other nonspatial features to construct a multiview similarity matrix *S*. The multiview similarity matrix incorporates both spatial and nonspatial views, enhancing the ability to capture complex structures in the dataset.

(12) $$\begin{array}{c} S=\alpha \cdot W_{s}+\left(1-\alpha \right) \end{array}$$

where *α* is a parameter controlling the balance between the spatial and other nonspatial views.

4. Thresholding: A threshold is applied to the outlier scores to identify samples with scores exceeding a predefined threshold as spatial outliers. In the implementation, the spatial weight matrix *W*_*s*_ is calculated based on the spatial distance matrix *D*. The multiview similarity matrix *S* is then constructed by combining the spatial weight matrix with other nonspatial views. Outlier scores are calculated for each sample, and a threshold is applied to identify spatial outliers in the crop yield prediction dataset [15–20]. The data sample is shown in Table 1.

## 5. Performance Analysis

**Figure 2:**
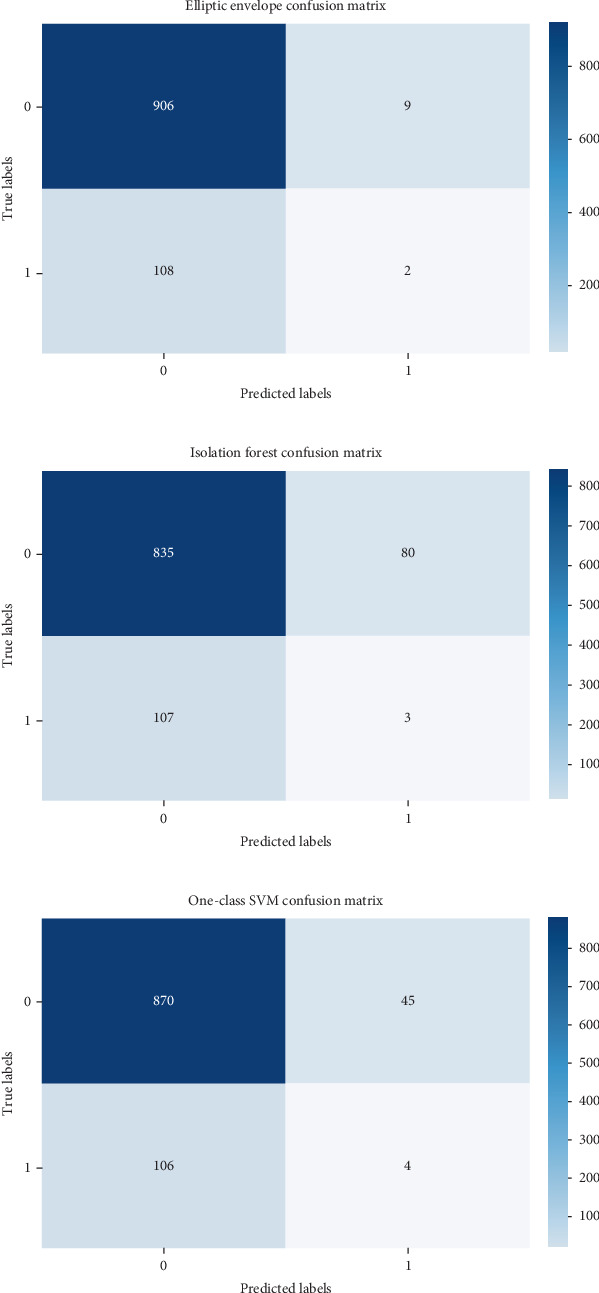
(a) Confusion matrix of elliptic envelope. (b) Confusion matrix of isolation forest. (c) Confusion matrix of one-class SVM.

**Figure 3:**
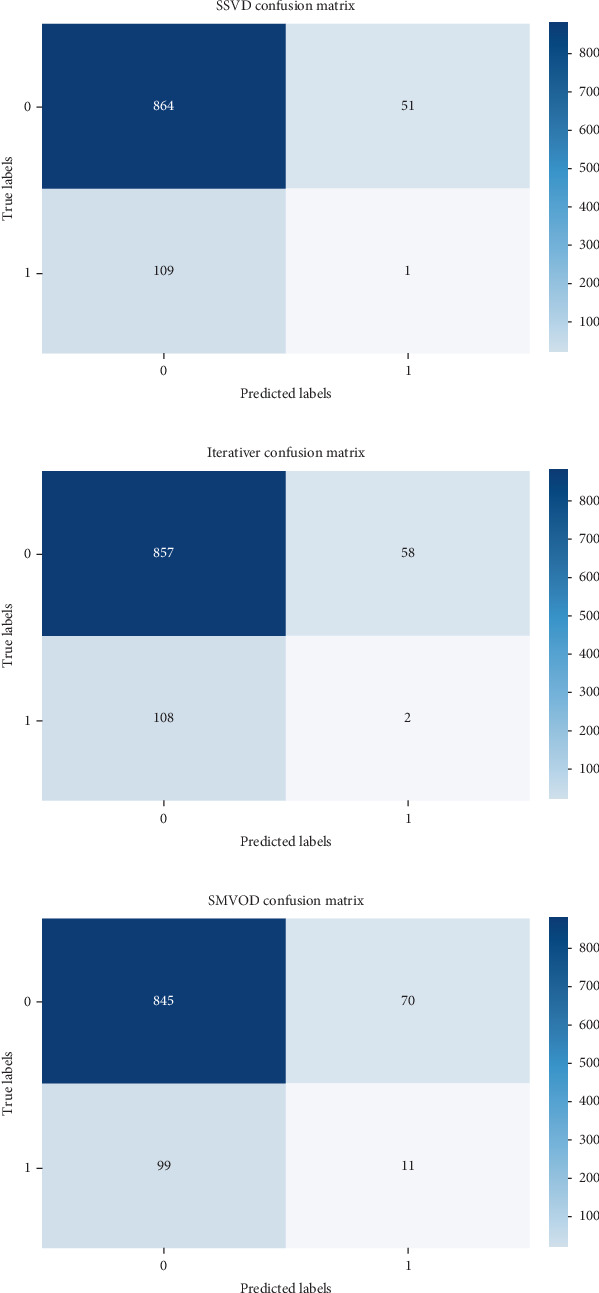
(a) Confusion matrix of SSVD. (b) Confusion matrix of iterative R. (c) Confusion matrix of SMVOD.

Evaluating performance through precision, recall, and *F*-score offers valuable insights into the efficacy of outlier detection methods. These metrics gain significance, especially in scenarios involving imbalanced datasets, where the presence of outliers is notably smaller compared to the abundance of inliers. The confusion matrix helps in assessing the performance of the algorithm by providing a clear breakdown of correct and incorrect classifications [21–25]. Figures 2 and 3 show the confusion matrix of the respective six algorithms which we used for outlier detection.

**Figure 4:**
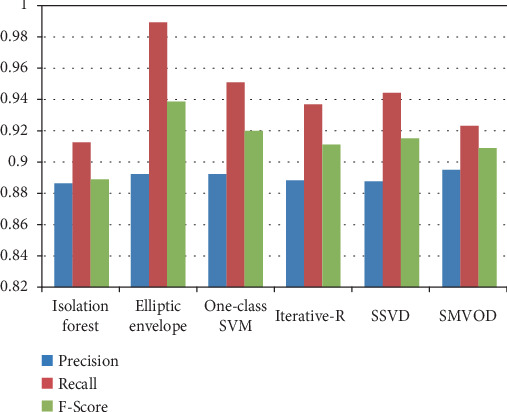
Performance analysis of all algorithms used for outlier detection.

According to Figure 4, all the outlier detection methods exhibit reasonable to high performance in terms of precision, recall, and *F*-score. Elliptic envelope and one-class SVM stand out with particularly high recall values, suggesting that they are effective in capturing a large proportion of actual outliers. Isolation forest, iterative R, SSVD, and SMVOD demonstrate well-balanced performance, providing reliable outlier detection with a reasonable trade-off between precision and recall.

## 6. Results

**Figure 5:**
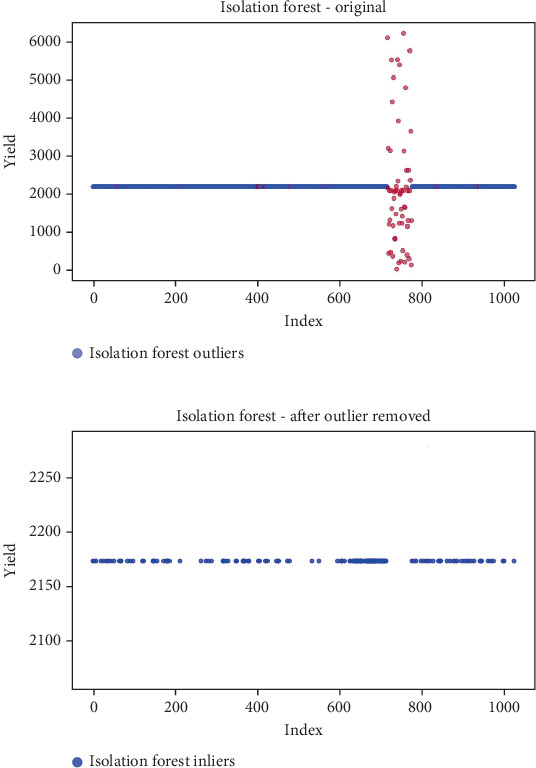
(a) Outliers in the data. (b) Outliers identified and removed using isolation forest.

**Figure 6:**
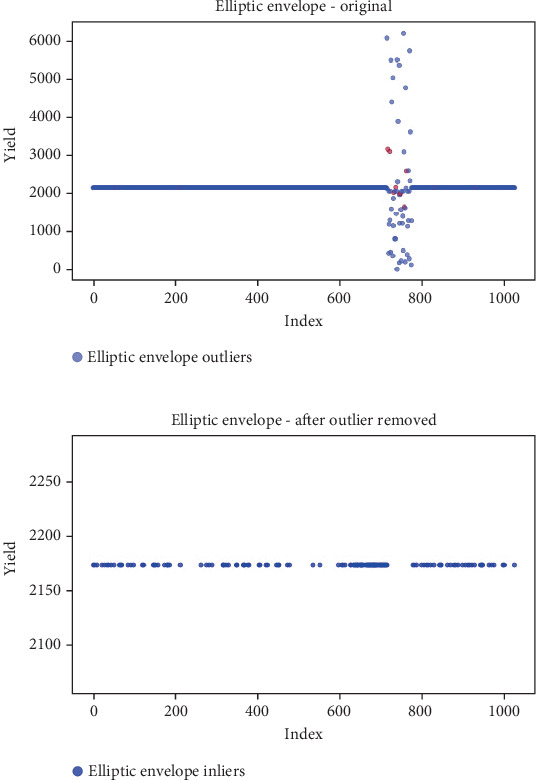
(a) Outliers in the data. (b) Outliers identified and removed using elliptic envelope.

**Figure 7:**
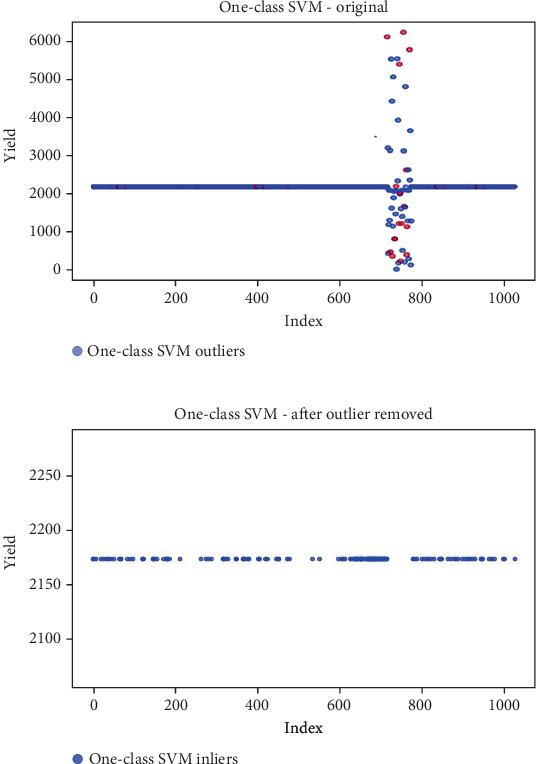
(a) Outliers in the data. (b) Outliers identified and removed using one-class SVM.

**Figure 8:**
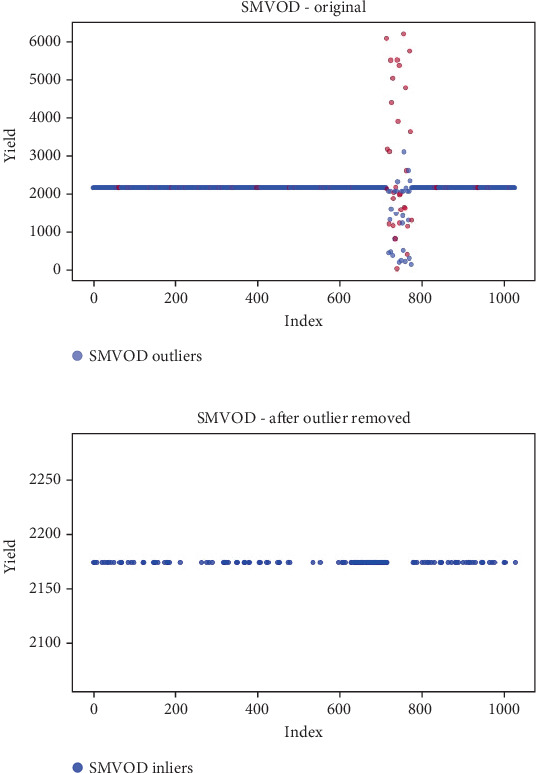
(a) Outliers in the data. (b) Outliers identified and removed using SMVOD.

**Figure 9:**
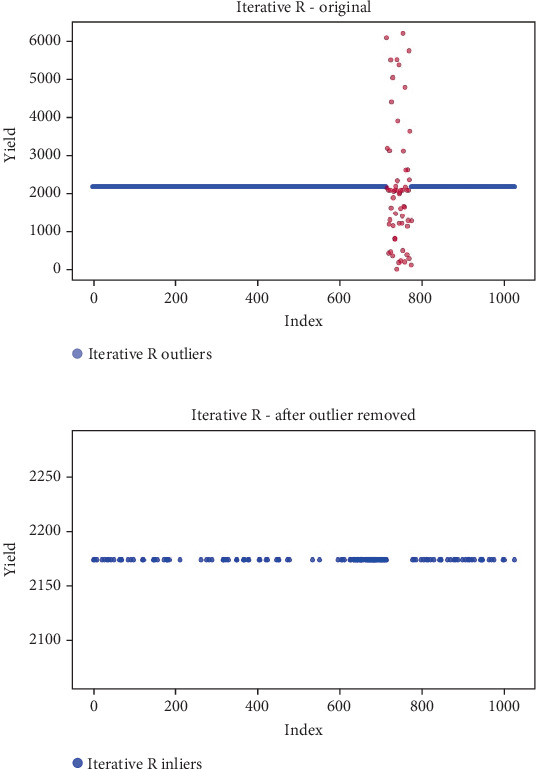
(a) Outliers in the data. (b) Outliers identified and removed using iterative R.

**Figure 10:**
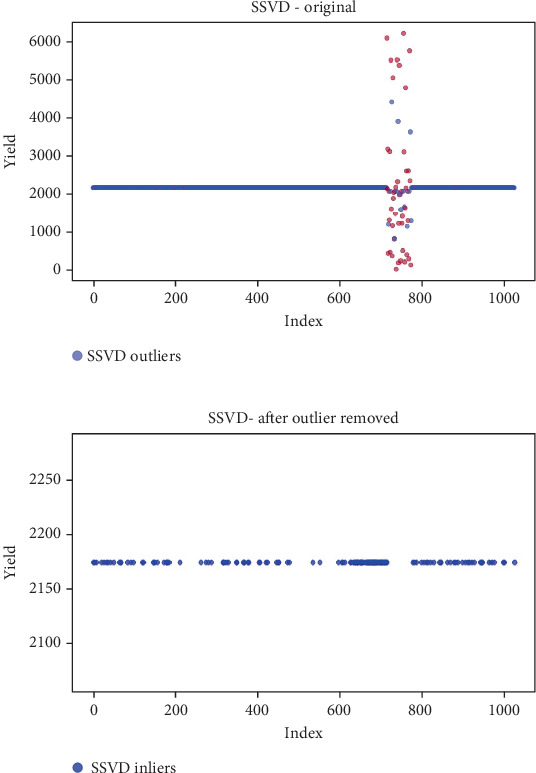
(a) Outliers in the data. (b) Outliers identified and removed using SSVD.

Before the implementation of an outlier detection algorithm, the dataset typically reflects the unprocessed distribution of raw data. At this stage, anomalies, noise, or outliers may be present, leading to irregular patterns or unexpected values in the dataset. Visualizations or figures generated from this raw data aid in illustrating the initial characteristics of the dataset. Figures 5a, 6a, 7a, 8a, 9a, and 10a depict the plot before the removal of outliers, showcasing scattered points with irregular patterns, and some observations deviate from the overall pattern [26–30]. Following the application of an outlier detection algorithm, the figures portray the dataset after processing, wherein outliers or anomalies have been identified and potentially eliminated. This step is pivotal for enhancing the data quality, particularly in scenarios where anomalies could adversely affect subsequent analyses or modeling. Figures 5b, 6b, 7b, 8b, 9b, and 10b illustrate the removal of outliers, resulting in a clearer and more representative pattern.

**Figure 11:**
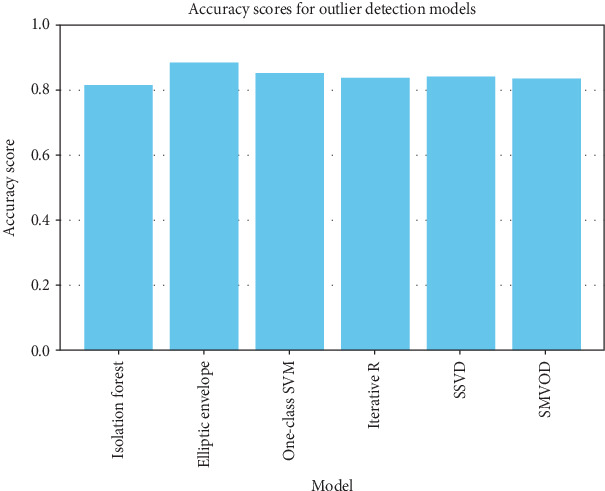
Accuracy of each outlier detection model.

The accuracy of each outlier detection model is depicted in Figure 11. Among these models, the elliptic envelope exhibits the highest accuracy at 91%, while the remaining models show accuracies ranging from 81% to 86%, with isolation forest registering the lowest accuracy [31–35].  Table 2 gives the measure of performance based on performance metrics like precision, recall, and *F*1 score of all algorithms.

## 7. Conclusion

This paper systematically explored and evaluated various outlier detection methods in the context of crop yield prediction for the Davangere region. Leveraging a diverse dataset from Taralabalu Krushi Vignyana Kendra, the study investigated isolation forest, DBSCAN, one-class SVM, elliptic envelope, iterative R, SSVD, and SMVOD. Each algorithm exhibited distinctive strengths and weaknesses, with elliptic envelope demonstrating the highest accuracy. Spatial methods showcased unique perspectives on outlier detection, while iterative R contributed to robust regression against outliers. Performance analysis using precision, recall, and *F*-score provided a comprehensive assessment of algorithm effectiveness, particularly valuable for imbalanced datasets. The visualizations illustrated the transformative impact of outlier removal on the dataset. Overall, this research contributes insights that enhance the understanding and application of outlier detection in the precision agriculture domain, supporting further research and development.

## Figures and Tables

**Table 1:** Sample dataset records.

**pH**	**EC**	**N**	**P**	**K**	**Lat**	**Long**	**Crops**	**District, year**	**Season**	**Temperature**	**Rainfall**	**Area**	**Production**	**Yield**	**Soil**	**pH**	**EC**
7.3	2.8	252	5.1	77.28	14.4212	76.5546	Jowar	DAVANAC 2015-201	Summer	24.4167	91.9167	19,327.6	62,160.7	2173.45	Sandy	7.3	2.8
7.64	0.5	273	7.69	93.02	14.4252	76.5226	Jowar	DAVANAC 2015-201	Summer	24.4167	91.9167	19,327.6	62,160.7	2173.45	Sandy	7.64	0.5
7.88	2.2	224	4.18	67.68	14.4004	76.4306	Jowar	DAVANAC 2015-201	Summer	24.4167	91.9167	19327.6	62160.7	2173.45	Loam	7.88	2.2
7.78	1.33	203	7.23	76.92	14.3342	76.1875	Cowpea	DAVANAC 2015-201	Kharif	24.4167	91.9167	19,327.6	62,160.7	2173.45	Clay loam	7.78	1.33
7.74	3.8	224	3.09	47.02	14.2341	76.1157	Maize	DAVANAC 2015-201	Kharif	24.4167	91.9167	19,327.6	62,160.7	2173.45	Maize	7.74	3.8
6.83	0.36	182	12.08	58.03	14.2286	76.1075	Jowar	DAVANAC 2015-201	Summer	24.4167	91.9167	19,327.6	62,160.7	2173.45	Clay	6.83	0.36
8.27	0.26	238	5.13	96.86	14.2197	76.1154	Paddy	DAVANAC 2015-201	Summer	24.4167	91.9167	19,327.6	62,160.7	2173.45	Clay loam	8.27	0.26
8.15	0.45	203	8.36	86.97	14.2005	75.7819	Maize	DAVANAC 2015-201	Rabi	24.4167	91.9167	19,327.6	62,160.7	2173.45	Clay	8.15	0.45
5.74	0.46	350	21.6	101.76	14.2149	75.7171	Paddy	DAVANAC 2015-201	Summer	24.4167	91.9167	19,327.6	62,160.7	2173.45	Clay	5.74	0.46
5.07	0.13	336	30.4	78.62	14.2252	75.7335	Paddy	DAVANAC 2015-201	Rabi	24.4167	91.9167	19,327.6	62,160.7	2173.45	Clay	5.07	0.13
8.52	0.32	182	5.41	87.31	14.2192	75.6752	Maize	DAVANAC 2015-201	Kharif	24.4167	91.9167	19,327.6	62,160.7	2173.45	Loam	8.52	0.32
8.12	0.16	259	6.8	102.67	14.3234	75.7593	Paddy	DAVANAC 2015-201	Rabi	24.4167	91.9167	19,327.6	62,160.7	2173.45	Red	8.12	0.16
8.3	0.28	273	6.17	57.64	14.308	75.7424	Cowpea	DAVANAC 2015-201	Rabi	24.4167	91.9167	19,327.6	62,160.7	2173.45	Red	8.3	0.28
8.9	0.22	210	5.08	47.85	14.3364	75.7416	Groundnut	DAVANAC 2015-201	Kharif	24.4167	91.9167	19,327.6	62,160.7	2173.45	Red	8.9	0.22
8.2	0.21	301	7.61	120.48	14.4354	75.7647	Groundnut	DAVANAC 2015-201	Rabi	24.4167	91.9167	19,327.6	62,160.7	2173.45	Sandy	8.2	0.21
8.49	1.2	280	7.1	67.68	14.3327	75.7777	Groundnut	DAVANAC 2015-201	Summer	24.4167	91.9167	19,327.6	62,160.7	2173.45	Sandy	8.49	1.2
8.28	0.42	245	4.81	91.1	14.3078	75.7611	Jowar	DAVANAC 2015-201	Rabi	24.4167	91.9167	19,327.6	62,160.7	2173.45	Clay loam	8.28	0.42
8.3	0.16	231	4.18	76.99	14.5853	75.6677	Groundnut	DAVANAC 2015-201	Summer	24.4167	91.9167	19,327.6	62,160.7	2173.45	Clay	8.3	0.16
7.27	0.48	224	13.16	119.66	14.6048	75.6487	Maize	DAVANAC 2015-201	Rabi	24.4167	91.9167	19,327.6	62,160.7	2173.45	Red	7.27	0.48
7.88	0.31	266	10.08	98.11	14.5125	75.9491	Paddy	DAVANAC 2015-201	Kharif	24.4167	91.9167	19,327.6	62,160.7	2173.45	Clay loam	7.88	0.31
7.8	0.41	294	9.17	50.64	14.5545	75.755	Groundnut	DAVANAC 2015-201	Kharif	24.4167	91.9167	19,327.6	62,160.7	2173.45	Sandy	7.8	0.41
8.18	1.25	231	5.03	104.68	14.5639	75.7905	Paddy	DAVANAC 2015-201	Rabi	24.4167	91.9167	19,327.6	62,160.7	2173.45	Red	8.18	1.25
7.6	0.45	203	13.77	55.1	14.5767	75.8214	Cowpea	DAVANAC 2015-201	Kharif	24.4167	91.9167	19,327.6	62,160.7	2173.45	Clay loam	7.6	0.45
7.9	4.8	245	7.68	77.42	14.5904	75.8366	Jowar	DAVANAC 2015-201	Kharif	24.4167	91.9167	19,327.6	62,160.7	2173.45	Sandy	7.9	4.8
7.88	0.31	266	10.08	98.11	14.5125	75.9491	Paddy	DAVANAC 2015-201	Kharif	24.4167	91.9167	19,327.6	62,160.7	2173.45	Clay loam	7.88	0.31

**Table 2:** Performance comparison of outlier detection algorithms based on precision, recall, and *F*1 score.

**Algorithm**	**Precision**	**Recall**	**F**1** score**
Elliptic envelope	0.8925	0.9891	0.9383
Isolation forest	0.8864	0.9126	0.8993
One-class SVM	0.8914	0.9508	0.9201
Iterative R	0.8881	0.9366	0.9117
SSVD	0.8880	0.9443	0.9153
SMVOD	0.8951	0.9235	0.9091

## Data Availability

The data that support the findings of this study are available from the corresponding author upon reasonable request.
